# Combined diffusion and perfusion index maps from simplified intravoxel incoherent motion imaging enable visual assessment of breast lesions

**DOI:** 10.1038/s41598-025-01984-2

**Published:** 2025-05-19

**Authors:** Petra Mürtz, Alois M. Sprinkart, Wolfgang Block, Julian A. Luetkens, Ulrike Attenberger, Claus C. Pieper

**Affiliations:** 1 Department of Diagnostic and Interventional Radiology, University Hospital Bonn, Venusberg-Campus 1, 53127 Bonn, Germany; 2 Department of Radiotherapy and Radiation Oncology, University Hospital Bonn, Venusberg-Campus 1, Bonn, Germany; 3 Department of Neuroradiology, University Hospital Bonn, Venusberg-Campus 1, Bonn, Germany; 4 Department of Biomedical Imaging and Image-Guided Therapy, General Hospital of Vienna (AKH), Medical University of Vienna, Waehringer Guertel 18-20, Wien, Austria

**Keywords:** Intravoxel incoherent motion, Breast neoplasms, Diffusion magnetic resonance imaging, Perfusion, Sensitivity and specificity, Medical research, Breast cancer

## Abstract

The aim was to evaluate visual breast lesion assessment using single binary index maps (IDf) in comparison to the use of combined regions of interest (ROI) analysis of estimated diffusion coefficient (D′) AND perfusion fraction (f′), which proved to be the best method in a previous simplified intravoxel incoherent motion DWI, if diffusion-weighted imaging (DWI) is used as stand-alone tool. IDf, was constructed voxel-wise from cut-off values of D′ and f′. The cut-off values, the data of 105 malignant and 86 benign lesions and the ROIs were re-used. For visual assessment, IDf was displayed as two-colour b800 overlay with red representing “malignant” and green “benign” voxels. A lesion was rated as “malignant”, if a red hot spot was found within translucent hyperintensity on b800, otherwise as “benign”. Intraindividual comparison of quantitative analysis and visual assessment of IDf showed comparable accuracy, both to each other and to combined ROI-analysis of D′ and f′ maps (0.927 vs. 0.937, *p* = 0.157, and 0.921 vs. 0.937, *p* = 0.157, respectively). Thus, visual assessment of IDf can replace combined ROI analysis of D′ and f′ without loss in accuracy enabling a considerable facilitation in clinical routine.

## Introduction

Diffusion-weighted imaging (DWI) with analysis of apparent diffusion coefficient (ADC) is increasingly used for breast lesion discrimination. As an adjunct to dynamic contrast-enhanced magnetic resonance imaging (DCE-MRI) it improved diagnostic specificity^1^. As stand-alone tool it provided unenhanced imaging for breast lesion assessment whereby diagnostic accuracy was lower or similar to that of DCE-MRI depending on image quality and lesion types included^2,3^.

Intravoxel incoherent motion (IVIM) analysis allowed the separation of diffusion and perfusion effects detectable with DWI by determination of “true” diffusion coefficient (D), perfusion fraction (f) and pseudodiffusion coefficient (D*)^4,5^. For DWI with analysis of D instead of ADC better accuracy was reached if used as add-on to DCE-MRI and as stand-alone tool,^6–8^. The reason is that malignant breast lesions often have low diffusion but high perfusion leading to an increase of ADC values being closer to values of benign lesions in contrast to D values^9–20^. For DWI with analysis of D and f in combination instead of D accuracy was not improved if used as add-on to DCE-MRI but was improved if used as stand-alone tool^7^. For DWI as stand-alone tool, the combined ROI analysis of D and f parameter maps was superior to ADC or D analysis in the assignment of complicated cysts, haematomas or other liquid-filled lesions/compartments, which often have low D values similar to malignant lesions but have low perfusion fraction whereby malignant lesion have perfusion hot spots^21,22^.

In conventional IVIM analysis D, D* and f were determined simultaneously by fitting algorithms. However, fitting procedures require a high number of b-values and thus relatively long acquisition times^23^ and may lead to unstable fitting results, poor reproducibility^24–27^ and unreliable parameter values of f and D* in tissue with low perfusion^28^ such as normal fibroglandular tissue and benign lesions^18,19,29^. For application in clinical routine, the so-called “simplified IVIM” approach is better suited than conventional IVIM analyses. Simpified IVIM approach is based on the assumption that pseudodiffusion component has essentially decayed to zero for b-values above a suitably high threshold. It uses explicit computation of numerically stable IVIM parameter estimations of D (D′), f (f′) and D* (D*′) in combination with a small number of b-values leading to improved stability of IVIM parameters and lower acquisition times compared to fitting procedures^7,8,28,30–33^.

ROI placement for quantitative analysis can be facilitated by the use of colour-coded IVIM parameter maps^27,34–36^ and the display as b0 overlay for morphological reference^30,31,33,37^. However, the determination of ROI-wise mean values of at least D and f parameters or their estimations D′ and f′, the comparison with cut-off values and the combination of the results is still inconvenient. The combined binary IDf index maps constructed voxel-wise from ROI-wise obtained from cut-off values of D′ and f′ IVIM parameter estimations and their display as two-colour b800 overlay for vital tumour identification are a convenient option, because lesions can be easily classified as malignant or benign by assessing the number of red voxel values within translucent hyperintensities^38^. Up to now, such index maps were only evaluated for liver lesions classifying a lesion as malignant if visual assessment identified a predominant number of voxels with red voxels and otherwise as benign.^38^. The combination rule and cut-off values have to be adapted to breast lesions, because in contrast to liver lesions, malignant breast lesions are indicated by higher f′ values instead of lower f′ values in combination with lower D′ values^9–20^ . The purpose of the present study was to evaluate the use of combined binary IDf index maps displayed as two-colour b800 overlay for visual assessment for breast lesion assessment using DWI as stand-alone tool and to investigate whether the inconvenient combined ROI analysis of D′ and f′ parameter maps can be replaced without worsen diagnostic accuracy.

## Methods

### Subjects

This retrospective study was approved by the local institutional review board of the University Hospital Bonn (No. 084/13). The study was conducted in accordance with the Declaration of Helsinki in its latest revised version. The need for informed consent was waived by the local institutional review board of the University Hospital Bonn. The patient population consisted of 126 female patients (age 54 ± 12 years, mean ± standard deviation; range 25–82 years) who were examined in the previous study^7^, which provided details on patient selection and examination procedures. All 191 breast lesions (86 benign, 105 malignant) were re-examined (Table 1) to investigate DWI as a stand-alone tool. All lesions had hyperintensity on the b800-DWI. 135 lesions had suspicious contrast enhancement (30 benign, 105 malignant). All lesions with suspicious contrast enhancement had hyperintense appearance on the b800-DWI. Inclusion criteria were: having one or more lesions suspected on b800 DWI or DCE-MRI, which all had a confirmed diagnosis (see below), and having not yet received a neoadjuvant therapy or radiation treatment. Patients were excluded if they had implants, if the lesions were less than 8 mm in size (to avoid partial volume effects), or if the quality of DWI was insufficient due to pixel misalignments. The diagnosis of lesions with suspicious contrast enhancement according to the morphologic and kinetic features defined in the Breast Imaging-Reporting and Data System (BI-RADS) MRI lexicon^39^ were established on the basis of histopathological examination according to the WHO classification of breast tumours^40^ or follow-up investigations with a minimum interval time of 12 months. The diagnosis of benign lesions with non-suspicious contrast enhancement was established by DCE-MRI and confirmed by ultrasound and follow-up.

**Table 1:** Overview of included lesion types.

Type of lesion	N
A) Benign lesions	86
Lesions with no or non-suspicious contrast-enhancement:	56
- Simple cyst	20
- Seroma after biopsy or surgery	3
- Complicated (haemorrhagic/proteinaceous) cyst	6
- Haematoma	7
- Normal fibroglandular tissue	20
Lesions with suspicious contrast-enhancement:	30
- Fibroadenoma	11
- Fibrocystic mastopathy	10
- Adenomyoepithelioma	1
- Syringomatous adenoma	1
- Intraductal papilloma	1
- Sclerosing adenosis	1
- Flat epithelial atypia	1
- Intramammary lymph node	4
B) Malignant lesions	105
Lesions with suspicious contrast-enhancement:	105
- Invasive carcinoma G1 (6 ductal, 1 tubular)	7
- Invasive carcinoma G2 (23 ductal, 16 lobular, 1 ductolobular, 1 ductal mucinous, 1 other)	42
- Invasive carcinoma G3 (34 ductal, 1 lobular, 2 mixed, 2 necrotic, 4 other)	43
- Invasive carcinoma with unknown grading (1 ductal)	1
- Ductal carcinoma in situ (1 G2, 9 G3)	10
- Intramammary lymph node metastases	2

*N* Number.

### Magnetic resonance imaging protocol

The data of a single-shot spin-echo echo-planar DWI variant with 4 b-values (0, 50, 250, 800 s/mm^2^) (Table 2) had been acquired in a previous study on a clinical 1.5 T MRI scanner before contrast injection^7^.

**Table 2:** Technical parameters of the diffusion-weighted imaging sequence.

Name	Value
FOV (RL × AP)/orientation	400 × 300 mm/transversal
Slice number/thickness/gap	29/4.0 mm/-1.0 mm
Matrix/pixel size	132 × 101/3.0 × 3.0 mm
Echo time	60 ms
Repetition time	2,116 ms
EPI factor/half-Fourier factor/SENSE factor	55/0.6/2
Diffusion gradients	Three orthogonal directions
Duration $\delta$/distance $\Delta$ of the diffusion gradients	22.6/31.9 ms
b-values (number of excitations)	0, 50, 250 s/mm^2^ (3), 800 s/mm^2^ (6)
Fat suppression method	STIR (inversion time = 180 ms)
Water-fat shift/bandwidth	7.1 pixel/30.4 Hz
Bandwidth in EPI frequency direction	2,203.5 Hz
Acquisition time	2:53 min:s

*AP* Anterior–posterior, *EPI* Echo-planar imaging, *FOV* Field of view, *RL* Right-left, *SENSE* Parallel imaging with sensitivity encoding, *STIR* Short time inversion recovery.

### Post processing and image analysis

**Fig. 1:**
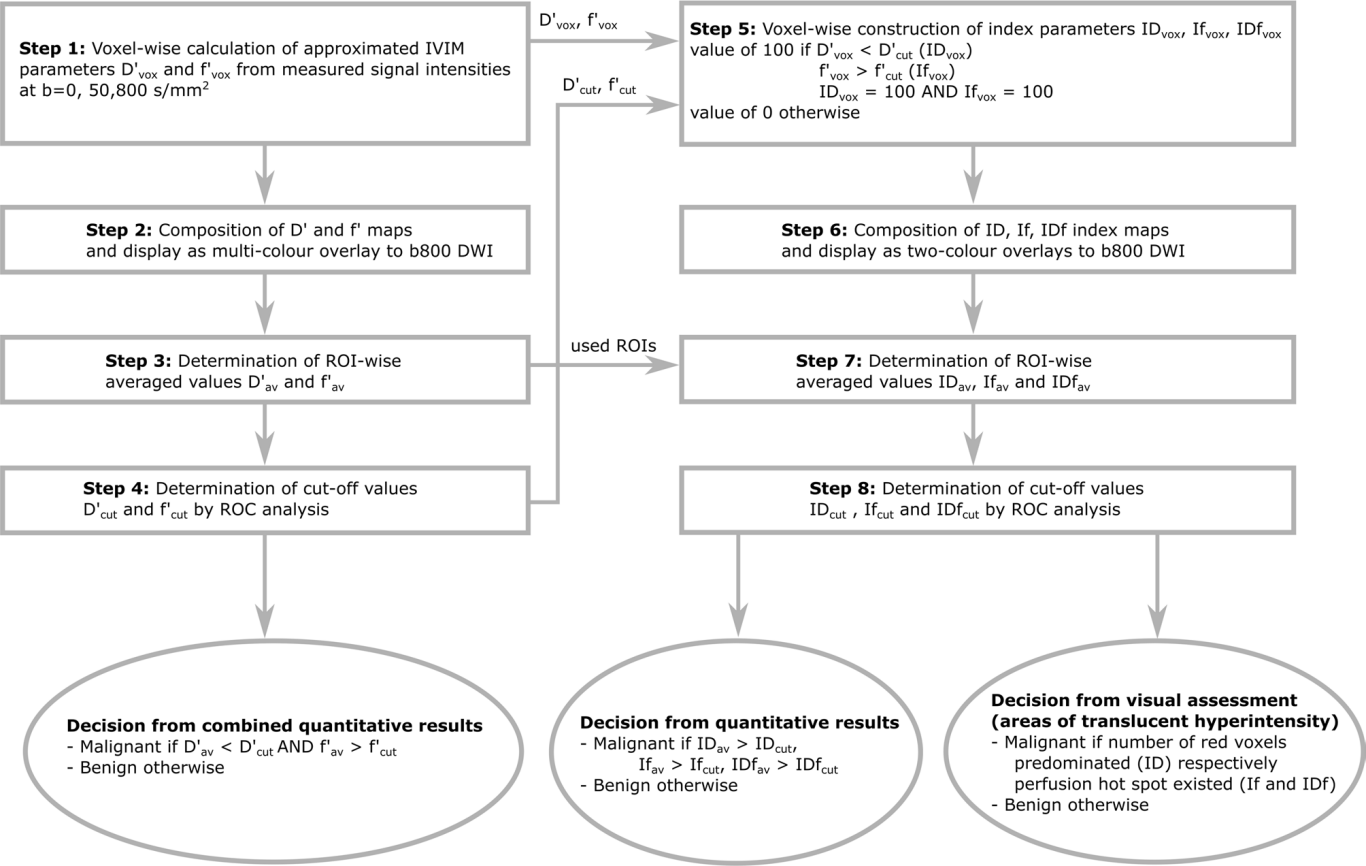
Flow diagram illustrating all postprocessing steps performed in both the previous study^7^ (left side) and present study (right side) to obtain the decisions.

The flow diagram given in Fig. 1 illustrates all postprocessing steps performed in the previous study^7^ (step 1–4) and in the present study (step 5–8) to obtain the decisions. All maps were calculated offline using a custom-developed MATLAB (MathWorks, Natick, MA, USA) software.

*Step 1*: In the previous study^7^, a two-compartment model of extravascular and intravascular space and a biexponential approach of the attenuation of signal intensity was assumed voxel-wise according to IVIM theory^4,5^:

1 $$\frac{{S_{vox} \left( b \right)}}{{S_{vox} \left( 0 \right)}} = f_{vox} \cdot e^{{ - b \cdot D^{*}_{vox} }} + \left( {1 - f_{vox} } \right) \cdot e^{{ - b \cdot D_{vox} }}$$

From signal intensities S_vox_ (b) and S_vox_ (0) detected at the three b-values b0 = 0 s/mm^2^, b50 = 50 s/mm^2^ and b800 = 800 s/mm^2^, D_vox_ and f_vox_ were estimated using the following formulas:

2 $$D^{\prime}_{vox} = ADC_{vox} \left( {b50,b800} \right) = \frac{{\ln \left( {S_{vox} \left( {b50} \right)} \right) - \ln \left( {S_{vox} \left( {b800} \right)} \right)}}{b800 - b50}$$

3 $$f^{\prime}_{vox} = f_{vox} \left( {b0,b50,b800} \right) = 1 - \frac{{S_{vox} \left( {b50} \right)}}{{S_{vox} \left( {b0} \right)}} \cdot exp^{{D^{\prime}_{vox} \cdot b50}}$$

*Step 2*: The voxel-wise determined D′_vox_ and f′_vox_ values were composed to parameter maps D′ and f′, respectively, which were displayed as multi-colour overlays to b800 DWI.

*Step 3*: By ROI-wise analysis of in D′ and f′ maps within perfusion hot spots (area of high perfusion and low diffusion and if not identifiable only in low diffusion) averaged values D′_av_ and f′_av_, respectively, were determined.

*Step 4*: By receiver operating characteristic (ROC) analysis of D′_av_ and f′_av_ values, cut-off values D′_cut_ (1229.2 × 10^−6^ mm^2^/s) and f′_cut_ (40.5 × 10^−3^) were determined.

Decision: From combined quantitative results of D′ AND f′ maps, a lesion was assigned as “malignant” if the D′_av_ value was lower than the D′_cut_ value AND the f′_av_ value was higher than the f′_cut_ value, otherwise as “benign”.

*Step 5*: In the present study, for voxel-wise construction of the index maps ID, If, and IDf the D′_vox_ and f′_vox_ values and the D′_cut_ and f′_cut_ cut-off values were re-used from the previous study^7^. The voxel value ID_vox_ was set to 100 (malignant voxel) if D′_vox_ was lower than D′_cut_, otherwise to 0 (benign voxel). The voxel value If_vox_ was set to 100 (malignant voxel) if f′_vox_ was higher than f′_cut_, otherwise to 0 (benign voxel). The voxel value IDf_vox_ was set to 100 (malignant voxel) if ID_vox_ AND If_vox_ were 100, otherwise to 0 (benign voxel).

*Step 6*: The voxel-wise determined ID_vox_, If_vox_ and IDf_vox_ values were composed to index maps ID, If and IDf, respectively, which were displayed as two-colour overlays to b800 DWI. Malignant and benign voxels were displayed on b800 overlay as red and green, respectively.

*Step 7*: By ROI-wise analysis of in ID, If and IDf index maps within the perfusion hot spots, which were re-used from the previous study^7^, averaged values ID_av_, If_av_ and IDf_av_, respectively, were determined. ID_av_, If_av_ and IDf_av_ values indicate the amount of “malignant” voxels within the ROI.

*Step 8*: By receiver operating characteristic (ROC) analysis of ID_av_, If_av_ and IDf_av_ values, cut-off values ID_cut_, If_cut_ and IDf_cut_ were determined.

Decision: From quantitative results of ID, If, and IDf, a lesion was assigned as “malignant” if the ID_av_, If_av_ and IDf_av_ , respectively, values were higher than the ID_cut_, If_cut_ and IDf_cut_, respectively, values, otherwise as “benign”. From visual assessment of ID, a lesion was assigned as “malignant” if the number of red voxels predominated in areas of translucent hyperintensity on b800 DWI, otherwise as “benign”. From visual assessment of If and IDf, a lesion was assigned as “malignant” if a red hot spot (an accumulation of red voxels but not along the edge of the lesion) existed in areas of translucent hyperintensity on b800 DWI, otherwise as “benign”.

### Method comparisons

The binarization during construction of index maps ID and If and the merging of the two maps into IDf index map (step 5) could both lead to less diagnostic accuracy compared to the combined analysis of the two parameter maps D′ and f′ (D′&f′)^7^. The reason is that malignant lesions are missed, if maximum perfusion is not in the area of minimum diffusion. Moreover, visual assessment of IDf could lead to less diagnostic accuracy than ROI-analysis of IDf, if lesion assessment is not definite in some lesions. Thus, the following method comparisons were performed.

The obtained area under the curve (AUC) values of I_Df_ were intraindividually compared to that of ID and to that of If. Sensitivity, specificity and accuracy together with the 95% confidence interval was calculated for IDf, ID and If. Diagnostic accuracy of IDf was intraindividually compared to that of ID, If, and D′&f′.

For visual assessment, the following two point scale was used to categorize the visual assignment as “definitely” or “probably”. The visual evaluation was performed by a physicist (P.M.) with more than 20 years of experience in DWI and by a radiologist (C.C.P.) with more than 15 years of experience in breast imaging. Reading was repeated by the first reader (P.M.) after 4 months. The reader were blinded to clinical information. Sensitivity, specificity and accuracy together with the 95% confidence interval was calculated for IDf, ID and If. Diagnostic accuracy of IDf was intraindividually compared to that of ID and If. Furthermore, the accuracy of visual assessment of IDf was intraindividually compared to that of quantitative analysis of IDf and of D′&f′. At last, from the visual assessment of the two persons and of the two visual assessments of one person, interobserver reliability and intraobserver reliability, respectively, were determined.

### Statistical analysis

According to the normal or non-normal distribution, continuous data are given as mean ± standard deviation or median and interquartile range (IQR, 25–75th percentile), respectively. The ROC analysis was performed using the pROC package in R (version 1.17.0.1, GNU project, Boston, MA, USA). To compare the area under the curve (AUC) values of dependent ROC curves, the DeLong method was applied^41^. Optimal cut-off values were calculated for maximum Youden’s index providing the highest combination of sensitivity and specificity. Sensitivity, specificity and accuracy were calculated as the ratio of the number of correctly assigned malignant lesions and the number of all malignant lesions, as the ratio of the number of correctly assigned benign lesions and the number of all benign lesions, and as the ratio of the number of correctly assigned lesions and the number of all lesions. Due to non-normal distribution, differences between the malignant and benign lesion groups (independent samples) was tested using the Mann–Whitney U-test and between analysis methods (dependent samples) using Wilcoxon test with statistical significance set as < 0.05 (SPSS, version 24.0, IBM, Armonk, NY, USA). The reliability of the visual assessment was determined by the intraclass correlation coefficient ICC_intra_ (repeated rating by the same investigator) and by the interclass correlation coefficient ICC_inter_ (rating by different investigators).

## Results

**Fig. 2:**
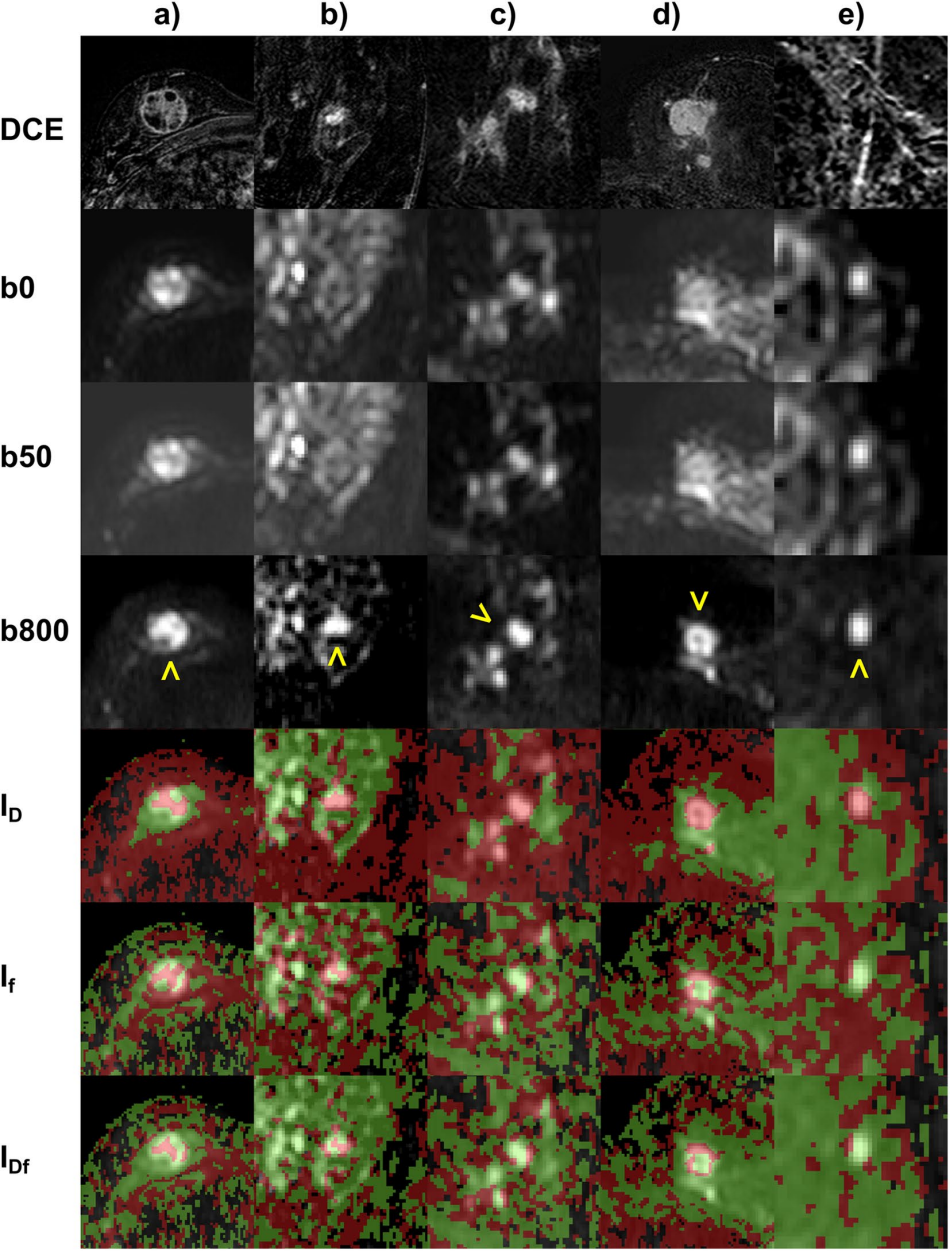
Typical examples of combined index maps IDf and index maps IADC, ID and If. Index maps were displayed as two-colour b800 overlay. Moreover, the related subtracted contrast-enhanced arterial phase T1-weighted images (DCE) and the original diffusion-weighted images with *b* = 0, 50, 800 s/mm^2^ (b0, b50, b800) are given. Lesions were assessed in the translucent hyperintense areas (marked in the b800 image with yellow arrowheads). For (**a**) an invasive ductal breast carcinoma (around 2.3 × 2.2 cm, grading G3), (**b**) an invasive lobular breast carcinoma (around 1.4 × 1.0 cm, grading G2), and (**d**) an invasive ductal breast carcinoma (around 2.3 × 2.2 cm, grading unknown), ID index maps show predominantly red voxels and If and IDf show clearly perfusion hot spots, indicating malignancy. For (**c**) a high grade lobular carcinoma in situ (LIN III, at least 5 cm in size, grading G2), ID index maps show predominantly red voxels and If and IDf show partially small perfusion hot spots at the edges (more than one row of red voxels), indicating also malignancy. For (**e**) a complicated cyst (around 0.8 × 0.6 cm), ID index maps show falsely malignancy, but due to the absence of any hot spots (only one row of red voxels at the edge do not count as hot spot), IDf classifies the lesion correctly as benign. In general, all liquid-filled lesions/compartments with low diffusion coefficient can be differentiated from malignant lesions by the uniformly low perfusion fraction.

**Fig. 3:**
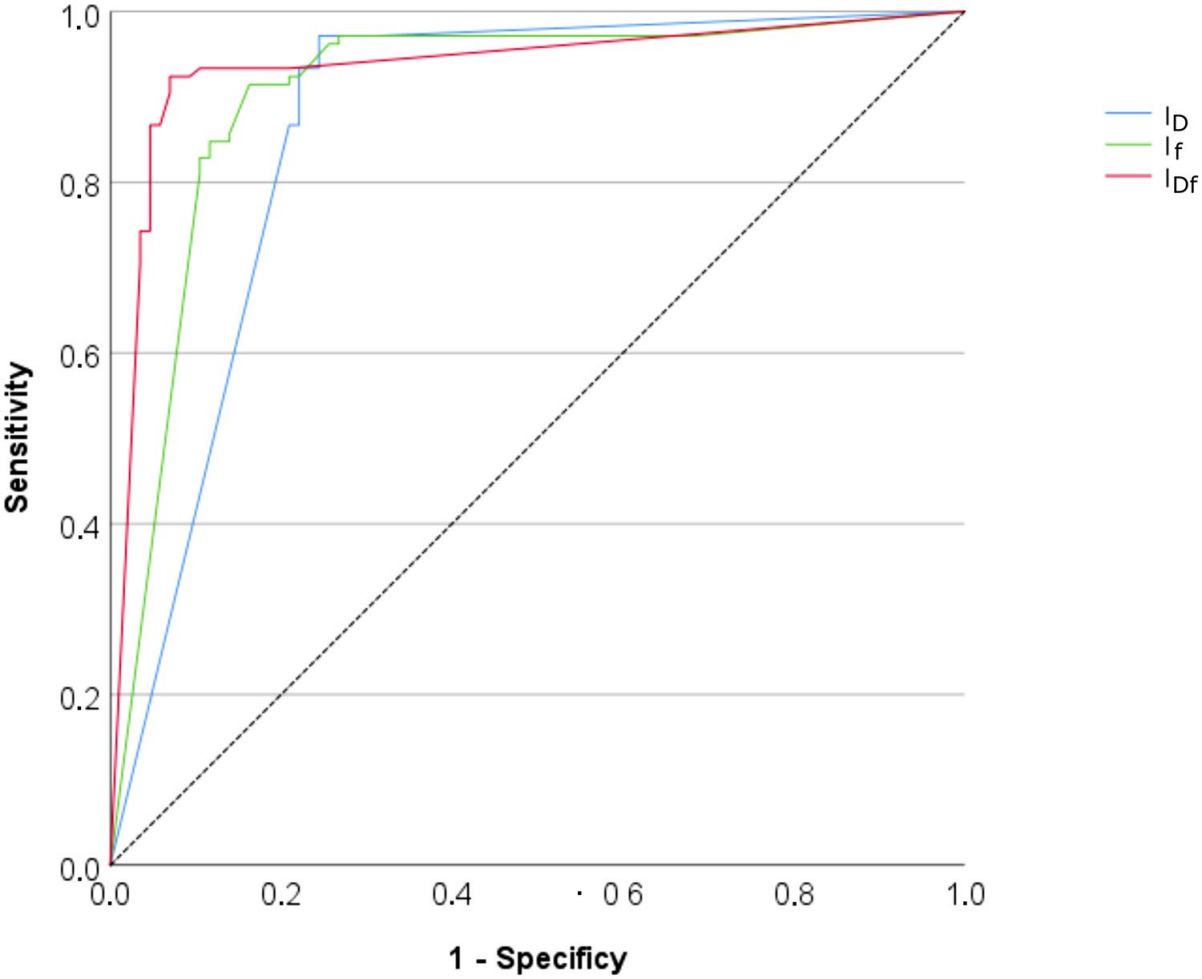
ROC curves obtained for IDf in comparison to ID and If. The area under the curve (AUC) is larger for IDf indicating better discriminability of the breast lesions.

Examples of DWI and index maps are given in Fig. 2. Quantitative analysis results are summarized in Table 3 and Fig. 3. The AUC value of IDf was larger than that of ID and If (*p* = 0.005 and *p* = 0.127, respectively). The diagnostic accuracy of IDf was larger than that of ID and that of If (*p* = 0.018 and *p* = 0.029, respectively). No significant differences were found between the accuracy of IDf and that of D′&f′ (*p* = 0.157).

**Table 3:** Results of the receiver operating characteristic analysis for the differentiation between benign (n = 86) and malignant (n = 105) breast lesions by ROI-analysis of indices IDf, ID, and If, whereby the analysed ROI-values indicate the amount of “malignant” voxels in the ROI.

Test var	Malign	Benign	Asym 95% CI	AUC	Cut-off point	Sen	95% CI	Spec	95% CI	Acc	95% CI
	MV ± SD	MV ± SD									
IDf	86 ± 28	8 ± 22	0.899–0.973	0.936	38.8 *	0.924	0.872–0.976	0.930	0.880–0.980	0.927	0.876–0.978
ID	91 ± 20	25 ± 41	0.800–0.915	0.857	37.5 *	0.971	0.938–1.000	0.744	0.658–0.830	0.869	0.803–0.935
If	92 ± 20	29 ± 34	0.860–0.951	0.906	73.2 *	0.914	0.859–0.969	0.837	0.765–0.909	0.880	0.816–0.944
D′&f′					1,229.2/40.5	0.943	0.898–0.988	0.930	0.880–0.980	0.937	0.889–0.985

The optimal cut-off point according the Youden index is given in 10–6 mm2/s for D′, in 10–3 for f′, and as percentages for IDf, ID, and If. Asymptotic significance of < 0.001 was reached for all parameter and index maps. *Acc* Accuracy, *AUC* Area under the curve, *Asym 95% CI* Asymptotic 95% confidence interval, *Malign* Malignant, *MV* Mean value, *SD* Standard deviation, *Sens* Sensitivity, *Spec* Specificity, *Test var* Test variable, * A higher test result indicates a more positive test.

In addition, sensitivity, specificity, and accuracy are given for each index and for D′ and f′ in combination (D′&f′)^7^.

By IDf 8/105 malignant and 6/86 benign lesions were falsely classified. By D′&f′ 6/105 malignant and 6/86 benign lesions were falsely classified^7^.

Visual assessment results are given in Table 4. The accuracy of IDf was higher than that of ID and that of If (*p* = 0.018 and *p* < 0.001, respectively). By IDf 7/105 malignant and 8/86 benign lesions were falsely classified. The assignment by IDf was “probably” in 8.9% (8 malignant, 9 benign lesions). The problem with “probably” assignments based on IDf was a difficult identification of perfusion hot spots when red voxels only occurred at the edge of a lesion. No significant differences were found between the accuracy of visually assessed IDf and that of quantitatively analysed IDf or that of quantitatively analysed D′&f′ (*p* = 0.705 and *p* = 0.317, respectively).

**Table 4:** Results of visual assessment of the index maps with respect to lesion malignancy.

Par	TN	FN	FP	TP	N	P	T	Sen	95% CI	Spec	95% CI	Acc	95% CI
Investigator 1													
I_D_	64	3	22	102	86	105	191	0.971	0.919–0.990	0.744	0.643–0.825	0.869	0.814–0.910
I_f_	34	1	52	104	86	105	191	0.990	0.948–0.998	0.395	0.299–0.501	0.723	0.655–0.781
I_Df_	78	7	8	98	86	105	191	0.933	0.869–0.967	0.907	0.827–0.952	0.921	0.875–0.952
Investigator 1 repeated after 4 months													
I_D_	63	3	23	102	86	105	191	0.971	0.919–0.990	0.733	0.631–0.815	0.864	0.808–0.905
I_f_	23	2	63	103	86	105	191	0.981	0.933–0.995	0.267	0.185–0.370	0.660	0.590–0.723
I_Df_	74	6	12	99	86	105	191	0.943	0.881–0.974	0.860	0.772–0.918	0.906	0.856–0.940
Investigator 2													
I_D_	62	3	24	102	86	105	191	0.971	0.919–0.990	0.721	0.618–0.805	0.859	0.802–0.901
I_f_	32	4	54	101	86	105	191	0.962	0.906–0.985	0.372	0.278–0.478	0.696	0.628–0.757
I_Df_	75	10	11	95	86	105	191	0.905	0.834–0.947	0.872	0.785–0.927	0.890	0.838–0.927

*Acc* Accuracy, *FN* False negative cases, *FP* False positive cases, *N* Number of benign cases (TN + FP), *P* Number of malignant cases (TP + FN), *Par* Parameter, *T* Total number of cases (N + P), *TN* True negative cases, *Sen* Sensitivity, *Spec* Specificity, *TP* True positive cases.

The repeated analysis by the same investigator and by the independent investigator (Table 4) revealed excellent intraobserver reliability ICCintra for IDf (0.950) and for ID and If in combination (0.949), and excellent interobserver reliability ICCinter for IDf (0.967) and for ID and If in combination (0.950).

## Discussion

The main results of the present study were: For DWI as stand-alone tool, IDf index maps reached better diagnostic accuracy than ID and If, for quantitative analysis and visual assessment of the index maps which were displayed as two-colour b800 overlay. Moreover, the accuracy of quantitative analysed and visual assessed IDf were comparable (0.927 vs. 0.921, *p* = 0.705) and reached both the accuracy of combined ROI analysis of IVIM parameters D′ and f′ which yielded best results in the previous study^7^ (0.927 vs. 0.937, *p* = 0.157, and 0.921 vs. 0.937, *p* = 0.157, respectively). Intraobserver and interobserver reliability of visual assessment of IDf index map was excellent. The results demonstrate that the single combined IDf index map can be used for the visual assessment of breast lesions when DWI is used as stand-alone tool. No additional malignant lesions were missed by visual assessment of IDf compared to combined ROI analysis of D′ and f′. The maximum perfusion was always within the area of minimum diffusion. More than 90% of the lesions could be definitively assigned. Thus, the inconvenient ROI-based analysis of D′ and f′ parameter maps can be replaced by the more convenient visual assessment of IDf index map when DWI is used as stand-alone tool. The search for a perfusion hot spot (an accumulation of red voxels but not along the edge of the lesion) on IDf displayed as two-colour b800 overlay can easily be performed in clinical routine.

In malignant breast lesions lower D and higher f values compared to benign lesions were found with conventional IVIM^9–20^ indicating higher cell density with reduced extracellular space and increased relative contribution of microvascular blood flow. It was found that perfusion in malignant breast lesions is increased in so-called hot spots and that higher accuracy in lesion discrimination can be reached using perfusion hot spot ROIs instead of vital tumour ROIs^7^. Breast cancers typically exhibit perfusion heterogeneity^42^. In some previous studies ROI-averaged signal analysis was used in combination with bi-exponential fitting, if signal-to-noise ratio was not sufficient for voxel-wise analysis^18,20,23^. However, voxel-wise analysis is necessary in order to select perfusion hot spots. Perfusion hot spots in areas of minimum diffusion are potentially the most active parts of the tumour (proliferating cellularity and abundant angiogenic neovascularity), where biopsies should be taken (see Fig. 1 of reference^17^). Thus, IDf index maps are of special interest to determine the optimal region for performing a biopsy because vital tumour area with perfusion hot spots and low diffusion^7^ can be easily detected as accommodations of red voxels within translucent hyperintense area. Moreover, angiogenesis is an important prognostic indicator of tumour growth, metastatic potential, and response to adjuvant therapies^43^. At last, the use of IDf, just like the use of the combination of D′ and f′, is important for accurate assignment of liquid-filled lesions/compartments, which is of special relevance for the use of DWI as a stand-alone tool^21,22^. When visually assessing the index maps IDf displayed as two-colour overlay to b800 DWI, it is only necessary to distinguish whether a red hot spot is present in the area of translucent b800 hyperintensity. The use of single combined IDf index map allows a rapid and easy image interpretation. A reliable assignment by visual assessment of IDf was possible in more than 90% of the lesions. The reason for uncertain assignments was that red voxels only existed at the edge of the lesion and identification of perfusion hot spots was difficult. By visual assessment, comparable diagnostic accuracy was reached as for ROI-based quantitative analysis.

A limitation might be that only experienced readers have rated. Another limitation might be that by simplified IVIM approach parameters D and f were only estimated using an approximation^7,30,31^. On the other side, the use of explicit formulas instead of fitting procedures enables a simple and stable determination leading to reliable information. It should be mentioned that in breast DWI it is important to use good fat-suppression such as short time inversion recovery (STIR) fat-suppression or spectral adiabatic (or attenuated) inversion recovery (SPAIR) after good B0 shimming^44^. STIR leads to better fat suppression homogeneity than spectral-selective methods^44–46^ and thus to less partial volume effects with unsuppressed fat signal being a problem due to large water-fat-shift in single-shot DWI^47^. For STIR, a better lesion detectability was found compared to spectral pre-saturation with inversion recovery (SPIR)^48^ but not compared to SPAIR^44^. For STIR, a larger measurement reproducibility of ADC was found compared to SPIR^47^. On the other hand, STIR is not suitable for use after the injection of gadolinium-based contrast agents^44,46^ and ADC values obtained with STIR may be over/underestimated depending on the T1 and ADC profile within tissue^44^. In contrast to fatty breasts, dense breasts did not affect lesion detectability and ADC values^49^. In general, DWI is not suited for patients with implants and in case of small lesions. Since the IVIM parameter f depends on the relaxation times, f may vary with field strength and sequence parameters used (especially b-values, echo times and repetition times)^50^, this also applies to the cut-off points used for the index maps.

In future studies, the visual assessment of IDf index maps displayed as two-colour b800 overlay should be evaluated as a stand-alone screening tool.

In conclusion, the inconvenient combined ROI-analysis of D′ and f′ parameter maps that are the gold standard when DWI is used as stand-alone tool can be replaced by the more convenient visual assessment of IDf index map displayed as two-colour b800 overlay without any loss in accuracy. The search for a perfusion hot spot as a sign of malignancy can be easy performed in clinical routine.

## Data Availability

The datasets generated during and/or analyzed during the current study are available from the corresponding author on reasonable request.
